# Neonatal head circumference by gestation reflects adaptation to maternal body size: comparison of different standards

**DOI:** 10.1038/s41598-022-15128-3

**Published:** 2022-06-30

**Authors:** Ruta Morkuniene, Janina Tutkuviene, Tim J. Cole, Egle Marija Jakimaviciene, Jelena Isakova, Agne Bankauskiene, Nijole Drazdiene, Vytautas Basys

**Affiliations:** 1 Department of Anatomy, Histology and Anthropology, Institute of Biomedical Sciences, Faculty of Medicine, Vilnius University, M.K. Ciurlionio str. 21, Vilnius, Lithuania; 2 UCL Great Ormond Street Institute of Child Health, London, UK; 3 Health Information Center, Institute of Hygiene, Didzioji str. 22, Vilnius, Lithuania; 4 Department of Human and Medical Genetics, Institute of Biomedical Sciences, Faculty of Medicine, Vilnius University, M.K. Ciurlionio str. 21, Vilnius, Lithuania; 5 Clinic of Children’s Diseases, Institute of Clinical Medicine, Faculty of Medicine, Vilnius University, Santariskiu str. 2, Vilnius, Lithuania; 6 Division of Biological, Medical and Geosciences, Lithuanian Academy of Sciences, Gedimino Ave. 3, Vilnius, Lithuania

**Keywords:** Anatomy, Health care, Medical research, Risk factors

## Abstract

Neonatal head circumference (HC) not only represents the brain size of *Homo sapiens*, but is also an important health risk indicator. Addressing a lack of comparative studies on head size and its variability in term and preterm neonates from different populations, we aimed to examine neonatal HC by gestation according to a regional reference and a global standard. Retrospective analysis of data on neonatal HC obtained from the Lithuanian Medical Birth Register from 2001 to 2015 (423 999 newborns of 24–42 gestational weeks). The varying distribution by gestation and sex was estimated using GAMLSS, and the results were compared with the INTERGROWTH-21st standard. Mean HC increased with gestation in both sexes, while its fractional variability fell. The 3rd percentile matched that for INTERGROWTH-21st at all gestations, while the 50th and 97th percentiles were similar up to 27 weeks, but a full channel width higher than INTERGROWTH-21st at term. INTERGROWTH-21st facilitates the evaluation of neonatal HC in early gestations, while in later gestations, the specific features of neonatal HC of a particular population tend to be more precisely represented by regional references.

## Introduction

Head circumference (HC) is a routine paediatric measurement that “acts as a proxy for brain size”^1^. Hence, HC at birth is an indirect measure of brain growth in utero that helps, in general, to evaluate foetal growth. Although at birth the human brain is 25% of its adult weight and continues growing until the age of approximately 10 years, HC is usually of interest primarily in infancy when the head growth velocity rate is maximal^1^.

Moreover, newborn HC, especially in those born preterm, is a significant health indicator. HC at birth and its postnatal growth dynamics are correlated with short-term and long-term health outcomes^2–8^. Greater HC at birth and faster postnatal head growth are associated with better neurocognitive and intellectual abilities in adolescence and young adulthood rather than birth weight per se^3–5^. In contrast, small HC at birth is related to the increased male risk of low intellectual performance^6^, emotional and behavioral disorders^8^, and higher arterial, especially systolic, blood pressure^9^. Considering neonatal HC an important health risk indicator for various periods of human development, an adequate HC growth assessment can facilitate not only the identification of infants at highest risk for long-term growth impairments, but also the choice of timely preventive health measures^10^.

Head size with its huge encephalisation ratio is the main characteristic of *Homo sapiens*. Thus it is likely to be more constant and less variable than other body size traits in a given population. On the other hand, postnatal growth is widely variable, and body size indices differ across geographic regions, populations^11,12^, or due to socio-economic circumstances^13–15^.

Thus tools for growth monitoring should be age- and sex-specific growth references or growth standards. Growth references describe how children from a particular region are growing, while growth standards present how children should grow under almost optimal conditions. The choice of growth reference for clinical practice is important for the evaluation of individual growth pattern. V. Neubauer et al.^16^ found that the interpretation of the postnatal growth of very preterm infants differed considerably depending on the four different references that were used: the proportion of microcephaly in very preterm infants varied from 3 to 25%. These distinct interpretations may lead to misdiagnosis and affect treatment and health monitoring strategies in clinical practice.

In 2006, the World Health Organization (WHO) published its child growth standards for children under five years. Subsequently the use of the WHO charts for particular countries or regions has been widely discussed^14,17^. In 2008, the International Foetal and Newborn Growth Consortium for the 21^st^ Century (INTERGROWTH-21^st^, IG-21) launched a multi-country project to develop similar prescriptive standards for foetal growth, neonatal size and postnatal growth of preterm infants^18,19^.

Many recent studies^11–13^ have considered the evaluation of postnatal growth in newborns. So far, there is a lack of studies comparing head size and its variability in term and preterm neonates from different populations and geographic regions. There is no clear evidence on whether global standards for newborn HC apply to neonates from all geographical areas. Moreover, the increasing prevalence of caesarean section in clinical practice, with fewer neonates born vaginally, described by M. Odent^20^ as a phenomenon of a sudden disappearance of the “evolutionary bottleneck”, may lead to increased variability in HC at birth.

In this context, the aim of the present study was to analyse HC in Lithuanian newborns according to their gestational age and sex and to compare the results with those provided by the IG-21 study and other countries with evolutionary insights on variability.

## Methods

### Study design and cohort selection

Our study examined the anonymized database from the Health Information Center of the Institute of Hygiene in Vilnius, Lithuania. The study was based on the Lithuanian Medical Data of Births registered from the year 2001 to 2015 and included all data on singleton liveborn newborns between 24 and 42 completed weeks of gestational age (GA), retrieved from medical records with the total duration of pregnancy in weeks. We excluded all cases of multiple births, stillbirth, undetermined gender, incomplete data (for sex, gestational age, birth weight, birth length, head circumference) or newborns with major congenital malformations and syndromes. The cases with the main newborn anthropometric indices (weight, length, head circumference) incompatible with gestational age (more or less than Mean (M) ± 3 Standard Deviations (SD) following the WHO standards^21^) were removed from the analysis. In total, the cohort sample size of 423 999 newborns was derived. The sampling procedure and exclusion criteria are presented in a flow diagram (Supplementary Fig. 1).

### Statistical analysis

The statistical analysis of data was performed using the standard statistical programs (SPSS 22.0, EXCEL, and R). The major parameters of descriptive statistics and percentiles of HC by GA and sex for Lithuanian newborns were calculated. The coefficient of variation (CV) was calculated and used in the comparative analysis with the foreign studies^22^.

GAMLSS was used to estimate the distribution applied to smooth the 3rd, 10th, 25th, 50th, 75th, 90th and 97th HC percentiles by GA and sex separately^23^. The LMST method (BCT distribution) were applied to the data obtained on each sex and each measurement, respectively. The resulting main percentiles (3rd, 50th and 97th) were compared with IG-21 from 24 weeks. The analysis was carried out using the GAMLSS package (version 4.3–3) of R 4.0.3 software (www.r-project.org).

The comparison of the present data on HC of Lithuanian neonates with the data provided by the IG-21 project was conducted. Both the published standards of IG-21 project^19,24^ were presented for both sexes for every gestational week and day separately (i.e. 30 + 0, 30 + 1), while GA of the present study was recorded as complete gestational weeks (i.e. 30, 31). Therefore, the comparative analysis of the present study with the IG-21 project by GA was made by comparing the mean of HC at the specific gestational week of IG-21. The differences between the means were calculated using *t*-test. A *p-value* of < 0.05 was considered to indicate a statistically significant difference.

The data were also expressed as sex and gestation specific Z-scores using IG-21 as reference.

### Ethics approval

The study was granted the approval of the governmental institution the Lithuanian Bioethics Committee (Permission No. 57, last addition—2017–02-06) and was performed in accordance with the relevant ethical guidelines and regulations.

## Results

The sample size of our study (Table 1) increased dramatically with gestational age from less than 50 neonates at 24 gestational weeks to nearly 100 000 at term for each sex. The mean HC of boys was 0.5–0.8 cm greater than for girls at every gestational week. Conversely the standard deviation (SD) and the coefficient of variation (CV) of HC fell steeply with gestational age (Table 1).

**Table 1:** Comparison of head circumference (HC) of Lithuanian newborns by sex and gestational age (GA) and the INTERGROWTH-21st (IG-21) reference^19,24^. n—count, M—mean, SD—standard deviation, CV—coefficient of variation, defined as standard deviation / mean.

GA (in weeks)	Present study				Intergrowth – 21st				Mean difference LT – IG-21
	n	M	SD	CV	n	M	SD	CV	
**Boys **									
24	28	23.0	1.0	0.043	3	22.7	1.6	0.070	0.3
25	71	23.6	1.4	0.059	10	23.6	1.6	0.068	0.0
26	89	24.5	1.4	0.057	13	24.5	1.6	0.065	0.0
27	124	25.5	1.5	0.059	12	25.4	1.6	0.063	0.1
28	211	26.6	1.7	0.064	19	26.3	1.6	0.061	0.3
29	190	27.5	1.7	0.062	19	27.2	1.6	0.059	0.3
30	303	28.5	1.7	0.060	25	28.1	1.6	0.057	0.4
31	306	29.7	1.7	0.057	37	28.9	1.6	0.055	0.8
32	533	30.6	1.6	0.052	52	29.8	1.6	0.054	0.8
33	744	31.5	1.6	0.051	33	31.1	1.3	0.042	0.4
34	1305	32.3	1.5	0.046	48	31.7	1.3	0.041	0.6
35	1977	33.0	1.5	0.045	127	32.2	1.3	0.040	0.8
36	3682	33.5	1.5	0.045	322	32.7	1.2	0.037	0.8
37	9651	34.3	1.5	0.044	848	33.2	1.2	0.036	1.1
38	24,745	34.9	1.4	0.040	2032	33.7	1.2	0.036	1.2
39	51,027	35.3	1.4	0.040	2985	34.1	1.1	0.032	1.2
40	93,843	35.5	1.4	0.039	2532	34.5	1.1	0.032	1.0
41	27,226	35.8	1.4	0.039	1147	34.9	1.1	0.032	0.9
42	987	35.8	1.5	0.042	204	35.2	1.1	0.031	0.6
**Girls**									
24	40	22.2	1.3	0.059	3	22.5	1.6	0.071	-0.3
25	65	23.1	1.3	0.056	7	23.4	1.6	0.068	-0.3
26	98	23.7	1.6	0.068	7	24.3	1.6	0.066	-0.6
27	122	25.0	1.7	0.068	11	25.1	1.6	0.064	-0.1
28	153	26.2	1.9	0.073	16	26.0	1.6	0.062	0.2
29	168	27.0	1.7	0.063	22	26.9	1.6	0.059	0.1
30	248	28.2	1.8	0.064	24	27.8	1.6	0.058	0.4
31	274	29.0	1.7	0.059	33	28.7	1.6	0.056	0.3
32	454	30.4	1.7	0.056	43	29.6	1.6	0.054	0.8
33	610	31.1	1.6	0.051	17	30.7	1.3	0.042	0.4
34	1066	31.9	1.5	0.047	65	31.3	1.2	0.038	0.6
35	1635	32.5	1.5	0.046	111	31.9	1.2	0.038	0.6
36	3183	33.1	1.5	0.045	293	32.3	1.2	0.037	0.8
37	8078	33.8	1.4	0.041	798	32.8	1.1	0.034	1.0
38	21,708	34.3	1.4	0.041	1783	33.2	1.1	0.033	1.1
39	48,487	34.8	1.4	0.040	2849	33.6	1.1	0.033	1.2
40	93,307	35.0	1.3	0.037	2486	33.9	1.1	0.032	1.1
41	26,354	35.2	1.3	0.037	1180	34.2	1.0	0.029	1.0
42	907	35.3	1.4	0.040	218	34.5	1.0	0.029	0.8

The mean HC of Lithuanian preterm and term newborns was greater than for IG-21^18,19^ from 31 weeks for boys and 32 weeks for girls, the difference increasing with gestational age (Table 1). The gestational age- and sex-adjusted Z-scores of HC based on IG-21^19,24^ showed the same pattern (Supplementary Fig. 2).

**Figure 1:**
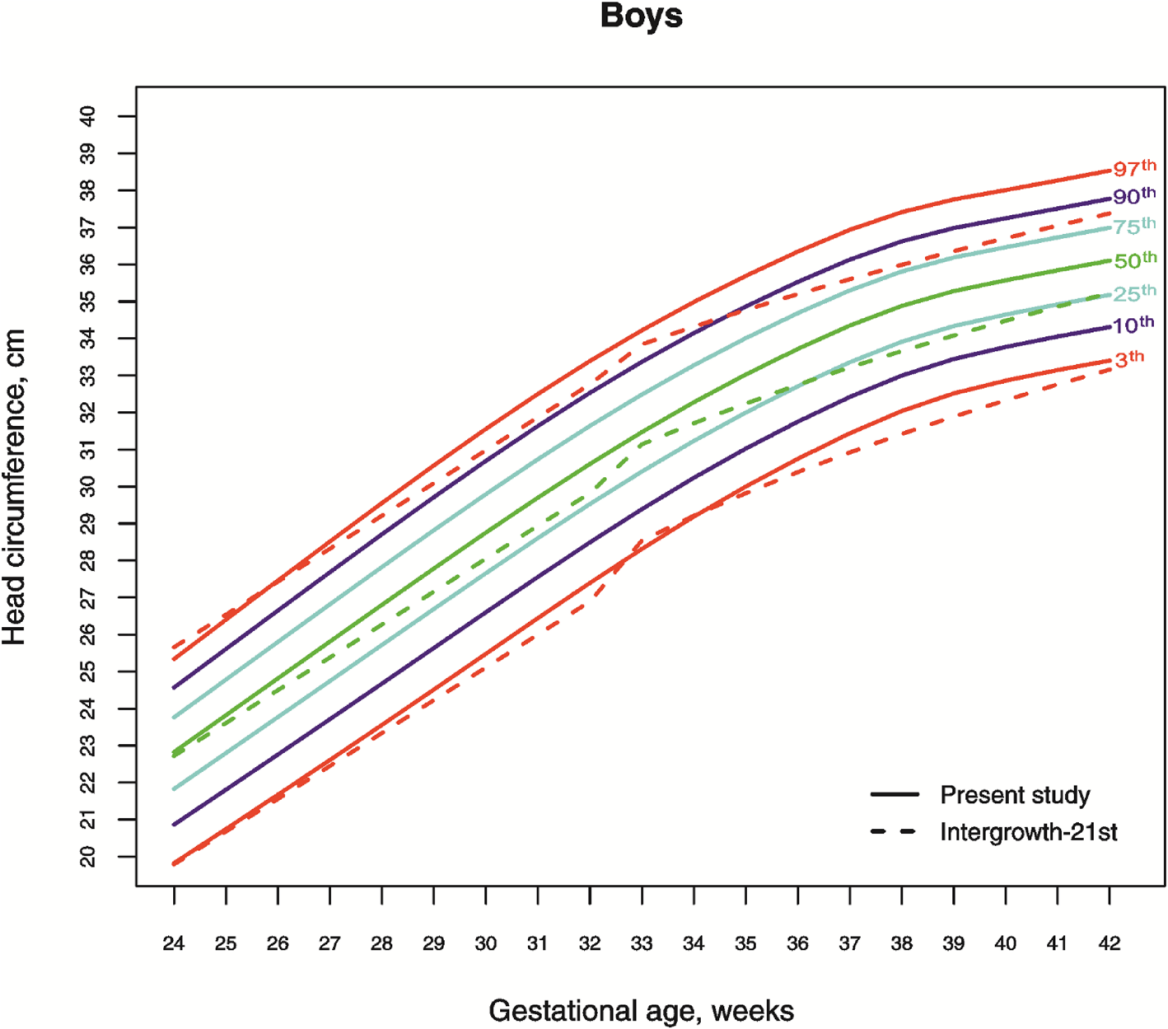
The 3rd, 10th, 25th, 50th, 75th, 90th and 97th smoothed percentile curves for head circumference (cm) in Lithuanian neonate boys and the 3rd, 50th and 97th percentiles for INTERGROWTH-21st^20,25^.

**Figure 2:**
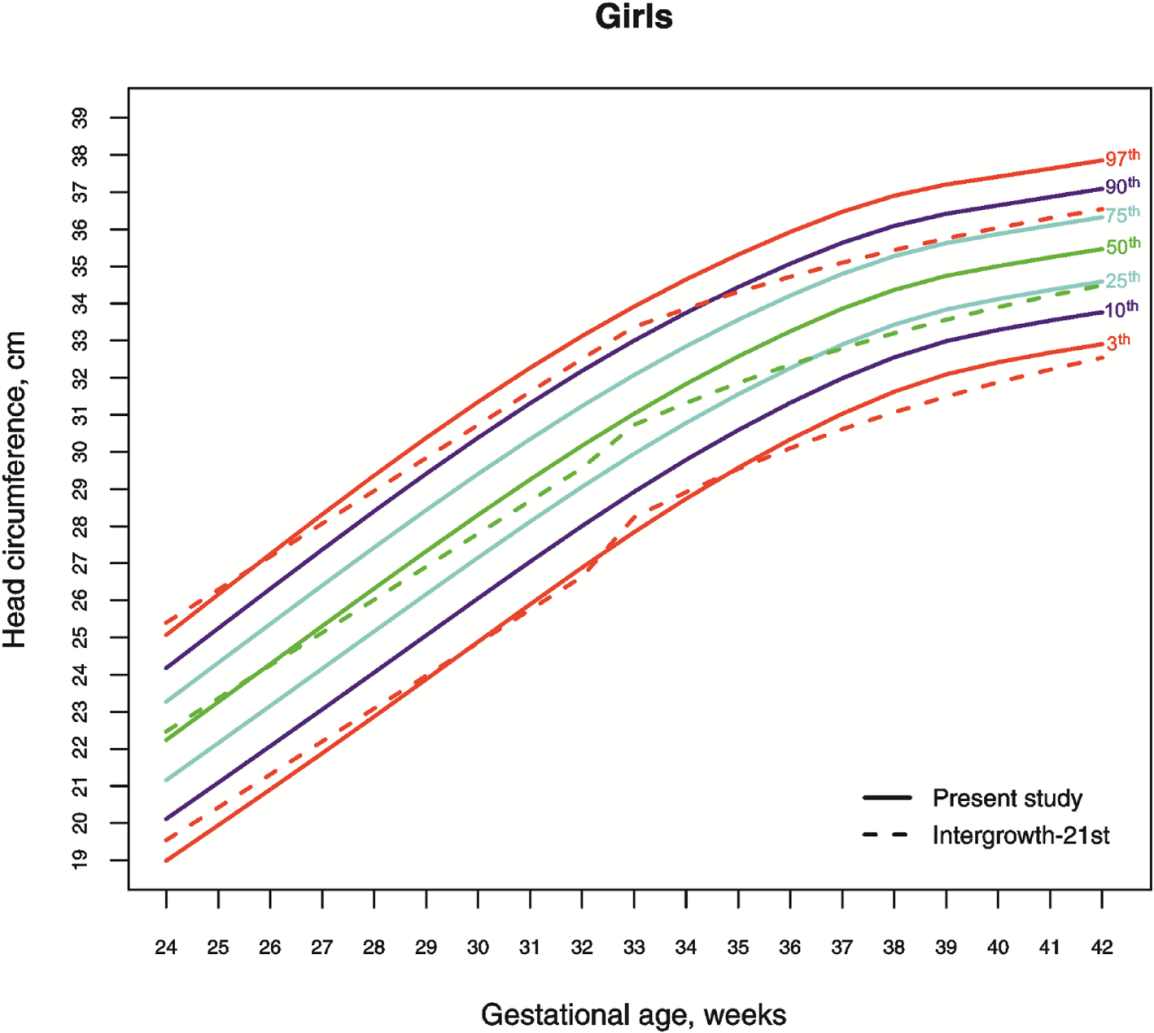
The 3rd, 10th, 25th, 50th, 75th, 90th and 97th smoothed percentile curves for head circumference (cm) in Lithuanian neonate girls and the 3rd, 50th and 97th percentiles for INTERGROWTH-21st^20,25^.

The 3rd, 10th, 25th, 50th, 75th, 90th, and 97th smoothed gestational age- and sex-adjusted percentile curves for HC of Lithuanian newborns are shown in Fig. 1 and 2. The variability of HC declines with increasing gestational age, and the negative skewness in the distribution is visible as wider gaps between the lower than the upper percentiles.

Comparing the 3rd, 50th and 97th Lithuanian HC percentiles by sex and gestation with those for IG-21 confirmed the pattern seen in Table 1, of close agreement at early gestations but a widening gap with increasing gestation, though restricted to the higher percentiles (Fig. 1 and 2). On the 3rd percentile, the differences in term newborns (gestation 37–40 weeks) amounted to 0.5–0.75 cm, falling to less than 0.5 cm in the post-term period. On the 50th and 97th percentiles, the differences varied from 1–1.5 cm (Fig. 1 and 2).

## Discussion

When monitoring the growth and development of neonatal HC, the primary concern is to use the best tools^10^. There is a lot of discussion recently concerning the choice of whether regional or global, age and sex-specific growth references or growth standards should be used for different populations^11–13^. Our study revealed that in late preterm and term periods, with a typically smallest neonatal head circumference (HC) variability within a population, the differences between populations are the most pronounced (Table 2). The differences between the findings of the studies examined increase with the increasing GA, and particularly starting from the late preterm period, and especially, in the term newborns. In the present Lithuanian study, the variation of the mean HC in extremely, moderate to late preterm newborns HC was < 1 cm, in term newborns—> 1 cm compared to IG-21 (Table 1). In extremely preterm gestations, the means of HC varies within most studies^25–28^ less than 0.5 cm compared to Lithuanian. However, according to some studies, in later gestations and in term newborns, the differences between populations in HC increase to more than 1 cm. Most of the similarities were found between Lithuanian and Finnish neonatal HC, the biggest differences – between Lithuania and Indonesia (Table 2).

**Table 2:** The comparison of neonatal head circumference (HC) of Lithuanian newborns by sex and gestational age (GA) and its coefficient of variation (CV) and the data provided by other studies^19,24–28^. SD—standard deviation, CV—coefficient of variation, defined as standard deviation/mean, N/A – not available.

GA (in weeks)	U. Sankilampi et al., FINLAND						Barbier et al., CANADA						Haksari et al., INDONESIA						Fok et al., CHINA					
	BOYS			GIRLS			BOYS			GIRLS			BOYS			GIRLS			BOYS			GIRLS		
	Mean	SD	CV	Mean	SD	CV	Mean	SD	CV	Mean	SD	CV	Mean	SD	CV	Mean	SD	CV	Mean	SD	CV	Mean	SD	CV
24	22.08	1.43	0.06	21.64	1.43	0.07	22.4	2.6	0.12	22.1	2.4	0.11	N/A	N/A	N/A	N/A	N/A	N/A	22.6	0.5	0.02	23.4	1.7	0.07
25	23.13	1.45	0.06	22.73	1.44	0.06	23.6	1.9	0.08	22.9	1.6	0.07	N/A	N/A	N/A	N/A	N/A	N/A	23.7	0.8	0.03	24.1	1.6	0.07
26	24.19	1.47	0.06	23.81	1.46	0.06	24.6	1.8	0.07	23.8	1.4	0.06	26.7	2.79	0.10	26.6	2.81	0.11	24.8	1	0.04	23.8	0.8	0.03
27	25.26	1.49	0.06	24.88	1.47	0.06	25.5	1.8	0.07	24.8	1.5	0.06	25.9	2.48	0.10	27	2.53	0.09	25.3	1.5	0.06	25	0.8	0.03
28	26.32	1.5	0.06	25.93	1.48	0.06	26.3	1.5	0.06	25.7	1.4	0.05	27.8	3.19	0.11	27.4	3.16	0.12	26.2	1.4	0.05	25.7	1.2	0.05
29	27.37	1.5	0.05	26.96	1.49	0.06	27.3	1.7	0.06	26.8	1.5	0.06	29	2.83	0.10	29.5	2.36	0.08	27.1	1.6	0.06	26.8	1.3	0.05
30	28.4	1.5	0.05	27.96	1.48	0.05	28.3	1.6	0.06	27.7	1.5	0.05	28.6	1.89	0.07	28.4	2.3	0.08	28.1	1.4	0.05	28.1	1.6	0.06
31	29.41	1.5	0.05	28.95	1.48	0.05	29.1	1.7	0.06	28.6	1.5	0.05	29.2	1.8	0.06	29.3	1.75	0.06	29	1.5	0.05	28.4	2	0.07
32	30.38	1.49	0.05	29.89	1.46	0.05	30.1	1.6	0.05	29.6	1.6	0.05	31.3	1.4	0.04	31.1	1.53	0.05	30	2	0.07	29.3	1.4	0.05
33	31.3	1.48	0.05	30.81	1.45	0.05	31.1	1.6	0.05	30.4	1.6	0.05	30.4	1.86	0.06	30.3	1.75	0.06	30.7	1.6	0.05	30.4	1.3	0.04
34	32.17	1.46	0.05	31.69	1.43	0.05	31.9	1.6	0.05	31.5	1.6	0.05	31	1.42	0.05	30.8	1.32	0.04	31.2	1.3	0.04	31.1	1.3	0.04
35	32.98	1.44	0.04	32.52	1.41	0.04	32.8	1.4	0.04	32.4	1.4	0.04	31.2	1.19	0.04	31.2	1.32	0.04	32.1	1.5	0.05	32.1	1.4	0.04
36	33.71	1.41	0.04	33.24	1.39	0.04	33.5	1.3	0.04	33.1	1.4	0.04	32.6	1.09	0.03	32.4	1.23	0.04	33.1	1.4	0.04	32.8	1.1	0.03
37	34.35	1.38	0.04	33.85	1.35	0.04	34.1	1.3	0.04	33.6	1.3	0.04	32.7	1.18	0.04	32.7	1.26	0.04	33.6	1.1	0.03	33.2	1.1	0.03
38	34.88	1.34	0.04	34.31	1.31	0.04	34.6	1.3	0.04	34	1.2	0.04	33.3	0.871	0.03	33.2	0.85	0.03	34.1	1.2	0.04	33.5	1.1	0.03
39	35.24	1.3	0.04	34.61	1.26	0.04	34.9	1.2	0.03	34.3	1.2	0.03	33.7	0.778	0.02	33.6	0.77	0.02	34.3	1.1	0.03	33.8	1.1	0.03
40	35.51	1.27	0.04	34.86	1.22	0.03	35.2	1.2	0.03	34.6	1.2	0.03	33.9	0.751	0.02	33.8	0.75	0.02	34.7	1.2	0.03	34	1.1	0.03
41	35.86	1.26	0.04	35.19	1.21	0.03	35.6	1.2	0.03	34.9	1.1	0.03	34.2	0.763	0.02	34.1	0.78	0.02	35	1.2	0.03	34.3	1.1	0.03
42	36.25	1.26	0.03	35.56	1.23	0.03	N/A	N/A	N/A	N/A	N/A	N/A	34.1	0.809	0.02	34	0.84	0.02	34.9	1.2	0.03	34.5	1.3	0.04

Analysing the variability of HC with regard to gestational age within and between populations, the coefficient of variation (CV) was examined. According to different studies^25–28^, the CV of HC in every population varies within a very narrow range, but is the highest in extremely preterm gestations, however, within the population, it decreases together with the increasing gestational age, same as the standard deviation (SD) (Tables 1 and 2). Hence, the closer to term, the narrower was the variability of the population’s neonatal HC. The CV of HC is higher in extremely preterm periods, but HC means and extremes appear to be very similar in different populations. We presume that in early gestation there is no need to strictly set head parameters according to the mother’s pelvis size, hence, greater biological variation is allowed, which is similar in most populations. On the other hand, the CV decreases with the increasing gestational age, but the means and marginal HC variants move according to a population-specific direction which is highly dependent on maternal size, particularly height and pelvic size^29^. Here, the size of the neonatal head seems to be maximally adapted to maternal pelvic size. These considerations support the idea that head circumference is strongly anthropometrically limited by the maternal bony pelvis—“evolutionary bottleneck”, as named by M. Odent^20^.

As the shape of the human pelvis is often interpreted as an evolutionary compromise between bipedal locomotion and childbirth of a highly encephalized neonate^30^, HC is expected to be more strongly genetically determined and anthropometrically limited by the indices of the bony birth canal. Even though the newborn HC should be less influenced by internal or external factors than birth weight or length, many studies have raised the discussion on the complex interaction between the intrinsic and extrinsic factors in the development of neonatal HC^31,32^. Furthermore, females with a large head, who are likely to give birth to neonates with a large head, were found to possess birth canals that are shaped to better accommodate large-headed neonates^29^. Moreover, it is already known that variation in the shape of the female pelvis is significantly geographically structured^33^.

**Figure 3:**
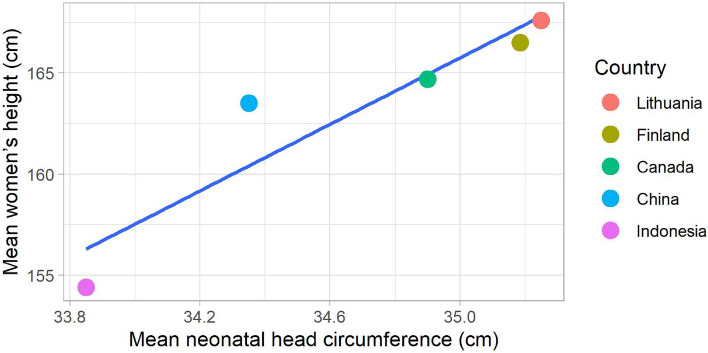
Neonatal head circumference (cm) at term (40 weeks gestation) in relation to average women’s height across countries^26–29,37^.

What is more, the pelvis shape was found to be significantly associated with the stature for taller women having a more oval pelvic inlet and better accommodating a larger foetal head^29^. In the study of R. G. Tague^34^ femoral length/stature in females showed a significant, positive partial correlation with the anteroposterior diameter and shape of the pelvic inlet. A recent Swedish study^35^ proved this relationship from the clinical point of view reporting decreasing risk of caesarean section (CS) with increasing maternal height after adjustment for maternal age, BMI, gestational age, parity, high birth weight and country of birth. With average Swedish women’s height of 166.1 cm, maternal height of 178–179 cm was associated with the lowest risk of CS (OR = 0.76, 95% CI 0.71–0.81), whereas height below 160 cm explained 7% of CS cases^35^. It is worth mentioning that according to the NCD Risk Factor Collaboration^36^, Lithuanian women are among the tallest women in the world with an average height of 167.6 cm. The comparative results of the female average height reflect the differences found between the mean neonatal HC from different populations, as shown in Table 2. Finnish women with an average height of 166.5 cm are closest to Lithuanians, followed by Canadians (164.7 cm), Chinese (163.5 cm), and finally Indonesians (154.4 cm)^36^. This supports the previous study’s findings^29^ that perhaps maternal height is linked to pelvic size, particularly the size of the birth canal, and through that to the neonatal HC. This possible relationship between neonatal HC (cm) at term (40 weeks of gestation) and average women’s height across compared countries is presented in Fig. 3 and compiled after^26–29,35^.

With regard to these findings, scientists debate the appropriateness of growth standards vs. references, regional vs. global for proper evaluation of growth and development of neonatal HC. In our study, most of similarities with global study of IG-21 were disclosed in cut-off points for the lowest percentiles in extremely preterm newborns, apart from that, extremely preterm newborns (especially girls) had more similarities not only in the third, but also in the 50th and 97th percentiles of HC. The Brazilian study^37^ revealed a similar pattern and found the trajectory of the third percentile parallel with the IG-21 study until the term period. The sample size of the IG-21^19^ reference was only modest for < 37 weeks gestation, and the later study on very preterm neonates ’should be interpreted with caution given the small sample size’^24^. This may explain a “wave” at 33 gestational weeks observed in the percentile curves of IG-21 (Figs. 1 and 2). Thus, although IG-21 facilitates the evaluation of the main HC percentiles for extremely preterm newborns and might serve as cut-off points for the pathological microcephaly in preterm newborns of different populations, it should be considered with caution to be confidently used as a global standard at early gestations.

As for the other extreme, the 97th percentile, above which infants would be diagnosed with macrocephaly, a large gap between the curves of both studies of more than 1 cm from late preterm to post-term was detected which could lead to an overestimation of macrocephaly in our cohort. If we compared our results with the HC curves provided by the Centers for Disease Control and Prevention (CDC)^38^, the gap would be smaller. In line with other studies^39^ evaluating the influence of growth curves used for the distribution of HC, our study also claims that the important consequences could have been triggered by the percentile misclassification. Therefore, from the standpoint of clinical practice, to predict the course of HC higher percentiles in moderate or late preterm periods and, especially, in Lithuanian term newborns, regional standards should be used.

Accordingly, the question has been raised by scientists whether children’s growth references should be global, or specific to different populations: ’it has become apparent that a single “global” reference fails adequately to mirror the diversity in human growth’^17^. Human growth is determined by inherited factors, and the significant variability of foetal growth in utero between ethnic groups supports this statement^31^. Therefore, the IG-21 project charts based on the idea that foetuses, infants, and children grow similarly all over the world under ideal nutritional, environmental, psychological living conditions have been widely discussed. A number of studies^32,37,40–46^ recently have compared their foetal and neonatal national growth references with the IG-21 study that was recently published, and obtained diverse results. Some studies did not find appreciable differences with IG-21 for newborn HC^40^ or a statistically significant difference was observed only of female HC in the 97th percentile^32^. While others determined that IG-21 standards for foetuses^47^ were found to be unrepresentative for regional populations leading to considerable overdiagnosis of foetal microcephaly or misclassification of infant birth size^37,41–46^ and a conclusion that regional validation was needed prior to the implementation of IG-21. We found that global standards like INTERGROWTH-21^st^ might facilitate the evaluation of neonatal head circumference in early gestations, while in later gestations, the specific features of neonatal head circumference of a particular population tend to be more precisely represented by regional standards.

Therefore, we suggest taking into consideration the regional standards for neonatal head circumference in order to better evaluate a possible clinical pathology. It is important to stress that over the process of evolution, neonatal body size and head circumference have adapted to the mother's body size, especially her pelvis, as a result of diverse adaptation mechanisms common to different populations in different geographical areas and under different living conditions.

## Conclusions

The closer to the late preterm—term period, the greater the differences between neonatal head circumferences in different populations. This threshold is slight, but it marks the inevitable influence of the evolutionary mechanisms that operate to first concentrate on vital biological capacities for neurodevelopment and only then allow genetics, ethnicity and other complex factors to influence the variability of neonatal head circumference.

Consequently, the global standards as IG-21 may serve for the evaluation of HC in early gestations, taking into account that most countries do not have a possibility to construct their own references due to small numbers of neonates born extremely preterm. In later gestations, regional standards more precisely represent the specific features of the neonatal HC of a particular population.

## Supplementary Information

Supplementary Information 1.

Supplementary Information 2.

## Data Availability

The data that support the findings of this study are available at the Health Information Center of the Institute of Hygiene of Lithuania, however restrictions apply to the availability of these data, used under a license for the current study, therefore they are not publicly available. The data are available from the corresponding author upon a reasonable request and with the permission of the Health Information Center of the Institute of Hygiene of Lithuania.
