# Supplementary material for: Neonatal head circumference by gestation reflects adaptation to maternal body size: comparison of different standards
Source: Sci Rep. 2022 Jun 30;12:11057. doi: 10.1038/s41598-022-15128-3 (PMC9246886; doi:10.1038/s41598-022-15128-3)
Supplement: Supplementary file 1 — Supplementary Information 1. [file 41598_2022_15128_MOESM1_ESM.docx]

**Boys**

**n=217042**

**Boys**

**n=226736**

**Girls**

**n=215763**

**Excluded cases of multiples**

**n=10292**

**n=428669**

**n=430755**

**n=431991**

**n=432207**

**Total sample size**

**n=442499**

**Girls**

**n=206957**

**Excluded cases of stillbirth or undetermined gender**

**n=1236**

**Excluded cases of +/- 3 SD**

**for HC**

**n=4670**

**Excluded cases with major malformations or syndromes**

**n=2086**

**Excluded cases of missing data**

**n=216**

**Final sample size**

**n=423999**

**Supplementary Figure 1** Flow diagram representing the sampling procedure and the exclusion criteria of the study. HC - head circumference, n – number, SD – standard deviation.
