# Supplementary material for: Neonatal head circumference by gestation reflects adaptation to maternal body size: comparison of different standards
Source: Sci Rep. 2022 Jun 30;12:11057. doi: 10.1038/s41598-022-15128-3 (PMC9246886; doi:10.1038/s41598-022-15128-3)
Supplement: Supplementary file 2 — Supplementary Information 2. [file 41598_2022_15128_MOESM2_ESM.docx]

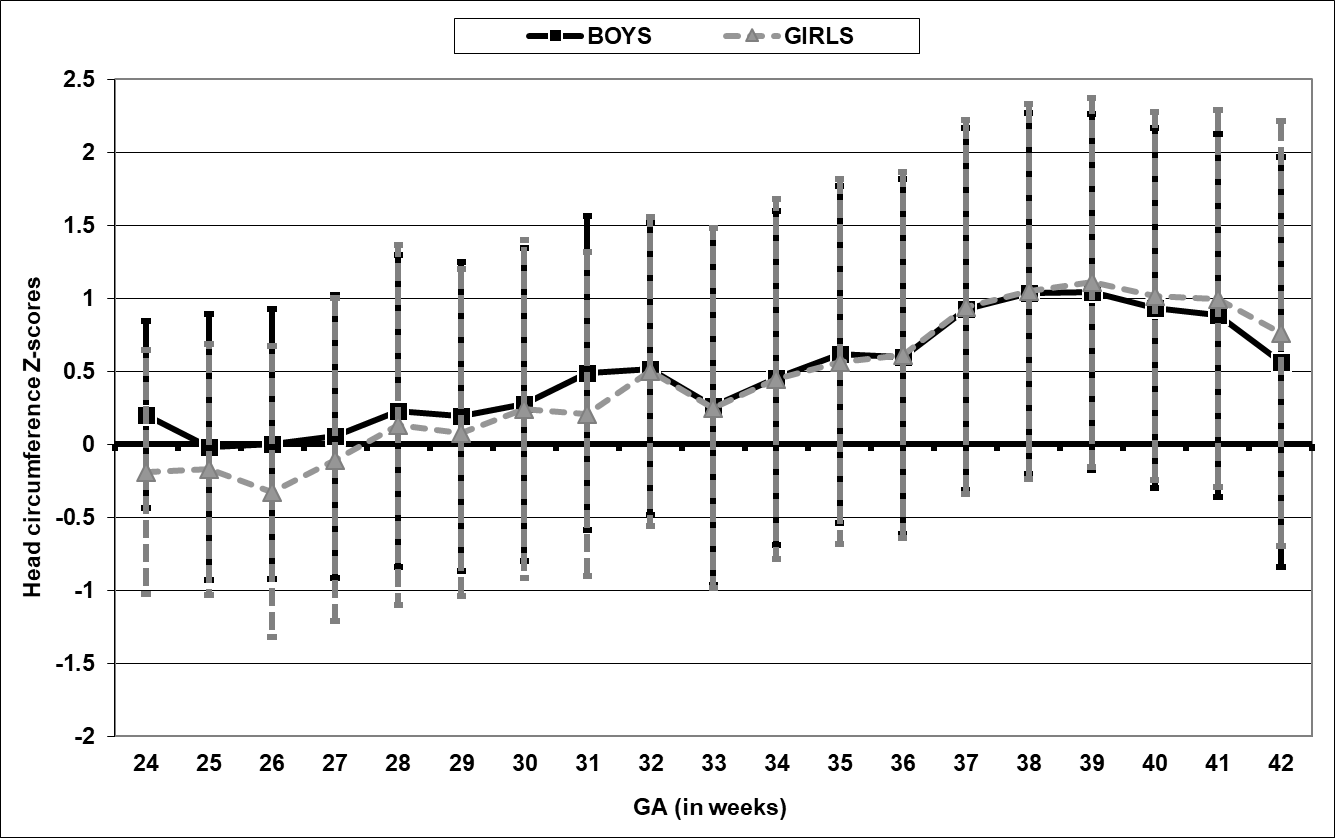


**Supplementary Figure 2** Z-scores for head circumference in Lithuanian boys and girls of 24 – 42 gestational weeks in relation to the INTERGROWTH-21^st^ study^20,25^. GA – gestational age.
